# Changes in thumb tapping rates and central motor conduction times are associated in persons with multiple sclerosis

**DOI:** 10.1007/s10072-022-05991-3

**Published:** 2022-04-04

**Authors:** Philipp Gulde, Mehmet Cetin, Joachim Hermsdörfer, Peter Rieckmann

**Affiliations:** 1 Center for Clinical Neuroplasticity Medical Park Loipl, Medical Park SE, Thanngasse 15, 83483 Bischofswiesen, Germany; 2 Chair of Human Movement Science, Department for Sport and Health Sciences, Technical University of Munich, Georg-Brauchle-Ring 60/62, 80992 Munich, Germany

**Keywords:** Multiple sclerosis, Central motor conduction time, Transcranial magnet stimulation, Motor evoked potential, Tapping, Prediction

## Abstract

**Introduction:**

In persons with multiple sclerosis, nerve conductivity can be reduced. The assessment is generally performed via motor evoked potentials (MEP). So far, a strongly associated motor performance surrogate for changes in the extracted central motor conduction time (CMCT) is missing.

**Methods:**

CMCT and performance in the nine-hole peg test and maximum thumb tapping frequencies over 10 s of 12 persons with multiple sclerosis were measured prior to and after training over 5 consecutive days. Each training consisted of 10,000 thumb taps at maximum effort with the dominant upper limb.

**Results:**

The dominant upper limb improved in maximum tapping frequency over 10 s (*d* = 0.79) and 10,000 taps (*d* = 1.04), the nine-hole peg test (*d* = 0.60), and CMCT (*d* = 0.52). The nondominant upper limb only improved in the nine-hole peg test (*d* = 0.38). Models of multiple linear regression predicted 0.78 (model 1, tapping performance as factors) and 0.87 (model 2, patient baseline characteristics as factors) of the variance in CMCT changes.

**Discussion:**

Changes in CMCT were well predictable, although the assessment of those surrogates is either not economic (model 1) or rather describing a potential of change (model 2). However, we were able to show moderate changes in CMCT within 5 days.

## Introduction

Tapping tasks have been shown to be a valid tool to assess disease severity and progression in multiple sclerosis (MS) [1–8] (expect in [9]), at least when participants are asked for maximum performance [1]. Commonly used dexterity tasks like the box-and-block or nine-hole peg test [10] are well associated with tapping performance [6, 8, 11], whereas such dexterity tasks can show low reliability [10] (but an intraclass correlation of 0.90 in [12]). Further, tapping tasks were strongly associated with central motor conduction time (CMCT) when MEP was recorded in stroke [13], which (the CMCT) is considered a good measure of disease progression in MS [2, 14].

CMCT can be impaired (slowed) by a loss of myelin due to MS [15] and since remyelination of lesions in MS patients has been shown in postmortem studies [16, 17], fostering such repair mechanisms are frequently the center of discussion, especially in terms of drug interventions [15, 18, 19]. While Armutlu et al. [20] were not able to observe changes in CMCT due to multidisciplinary rehabilitation programs in MS, increases in MEP amplitudes (and task performance) have been observed in healthy subjects in short term [21] as well as mid- to long-term after extensive tapping training [22]. Although many factors that influence neuroplastic adaptations will need to be considered—age [18, 23], disease progression [2], type of MS [6], reduced neuroplastic capacity [24], inflammation [19], and maladaptive neuroplasticity [25]—an improvement of CMCT by a highly repetitive training seems possible, as contrast-vision thresholds of MS patients suffering from chronic impairments due to optic neuritis improved directly (comparable to the changes in MEPs in Arias et al. [21] and several days after highly repetitive color stimulation [26].

If changes in CMCT can be assessed by tapping tasks, it would allow a quick, temporally dense, and economic assessment of a myelination surrogate in MS patients and could therefore support medical and pharmacological research that aims to foster remyelination or improve motor conduction. A prior study observed slowing of CMCT and disability progression in persons with progressive MS but did not correlate CMCT and motor performance [27]. Mamoei et al. [28] found correlations between the timed 25-ft walk test and CMCT of the lower limbs but not between the nine-hole peg test and upper-limb CMCT. Therefore, a sensitive upper-limb surrogate is still lacking. It is important to note that tapping task performance is dependent on many factors [5], including the complex interplay of a variety of brain regions. Therefore, the main target is not to search a surrogate for the CMCT but to examine the feasibility of tapping tasks to assess short- to midterm changes of the CMCT.

Our objective was to examine whether changes in CMCT of MS patients are reflected by the performance of a simple thumb tapping task. We hypothesized that highly repetitive (Gulde 2020), maximum [1] thumb tapping [22] training can change CMCT and motor performance, with a non-CMCT related (skill) transfer to the non-trained hand [29] and that changes in CMCT are associated with changes in maximum tapping performance. This would allow quick and economic estimations of the disease status.

## Methods

### Sample

A necessary minimum sample size of 12 participants was calculated on the basis of the coefficient of correlation between CMCT and tapping of Cakar et al. [13], one-sided with an *α* of 0.05, and a power of 0.80, using G*Power (version 3.1.9.7) [30].

We recruited 15 MS patients at the Center for Clinical Neuroplasticity Medical Park Loipl (Medical Park SE), a specialist clinic for neurology in Germany, of which 12 completed the study. Patient characteristics are given in Table 1. Two of the patients were current regular smokers (17%), one was underweight (BMI = 15.2 kg/m^2^; 8%), and six were overweight or obese (grade one) (BMI > 25.0 kg/m^2^; 50%). The average BMI was 24.8 kg/m^2^ ± 5.6 kg/m^2^. Of those patients, eight reported receiving disease-modifying therapies (67%).

**Table 1:** Patient characteristics as mean, standard deviation, and range, or quantity, respectively

Age in [a]	Expanded disability status scale (EDSS)	Time since patient-reported first manifestation in [a] (FM)	Sex	Type of MS
41.7 ± 10 (23–56)	3.5 ± 1.7 (2.0–8.0)	12.0 ± 8.7 (1–29)	58% female (7) 42% male (5)	9 relapsing remitting (75%) 3 prim./sec. progr. (25%)

Ethical approval was given by the ethics committee of the Medical Faculty of the Technical University of Munich (Germany). The study was conducted in accordance with the 1964 Declaration of Helsinki. All participants gave written informed consent to participate in the study.

### Parameters

In addition to the patient characteristics, the performance of the nine-hole peg test (PEG) in seconds was assessed for both hands in order to check for transfer of tapping training to fine motor control/object manipulation. Further, the maximum tapping rate of each thumb over the course of 10 s was assessed twice, and the best performance in Hz was noted (10 s TAP). Tapping speed was assessed using custom software for a Lumia 550 smartphone (Microsoft Corp.; programmed using Visual Studio 2017 C#, Microsoft Corp.). The number of recognized button-presses (approx. 5.5 cm × 5.8 cm) during a timer-set interval of 10 s was counted, and the average tapping frequency was computed. Patients held the smartphone in landscape orientation in both their hands. The CMCT in [ms] was computed on the basis of MEPs by a transcranial magnetic stimulator (MagPro R20, MagVenture Corp.), which is part of the clinic’s standard protocol for MS. The musculus adductor pollicis was used as the target muscle, and CMCT was calculated as the mean difference between central (M1) and peripheral MEP latencies of three trials (in case of deviations of more than 1 ms from the next latency, measures were considered invalid). A single coil (C-100, MagVenture Corp.) was used, and patients were seated in a chair with arm and leg support. The EMG signal was derived from surface electrodes and using a Neuropack μ device (Nihon Kohden Europe GmbH, Germany). For the central stimulation, we asked patients to slightly contract their muscles (pinching index finger and thumb together “like holding a fly between your fingers”). For the peripheral stimulation, we asked patients to relax their muscles. The intensity of the stimulations was set by increasing them step by step until a slight muscle contraction became visible. We decided against the protocol of 5/10 visible EMG reactions to keep the number of stimulations for patients at a minimum. As mentioned before, for the central and the peripheral stimulations, the mean of three successful stimulations was used to derive the CMCT. We decided to not include the MEP amplitude due to its reported very low intraclass correlation of 0.01 to 0.34 [31]. For each of the training sessions, the average tapping frequency was noted (10,000 TAP) in Hz as well as the needed time to complete the trial of 10.000 taps.

### Training

Patients were asked to perform one trial of 10,000 thumb taps as fast as possible with their dominant hand (11 right handed, 1 left handed) on each of 5 consecutive days. We decided to use the dominant (or in case of strong impairments of the dominant upper-limb the better-functioning thumb) in order to ensure an already well-developed cortical representation and the motor and motivational capacity to execute the task. The experiment was conducted with custom software (Visual Studio 2017 C#, Microsoft Corp.) on a Lumia 550 smartphone (Microsoft Corp.), showing the participant the number of taps (i.e., the progress), the duration in minutes and seconds (that ends up to be the trial duration), the mean frequency of the last 50 taps and the mean frequency of all taps. Additionally, a graph was showing the course of the mean of 50 taps (from left to right). Patients held the smartphone in landscape orientation in both their hands (like a joypad or Gameboy).

### Procedure

Patients were assessed with the nine-hole peg test (PEG), a maximum thumb tapping task over 10 s (10 s TAP), and their CMCT was computed by MEPs on days 1 and 6 (all with/for the dominant and nondominant hand). On day 1, they also started with their first training of 10,000 taps with the thumb of their dominant hand right after the assessment. The 10-s maximum tapping rate (10 s TAP) of both thumbs was assessed on all days but day 3 and day 4 (weekend), when patients were training in an unsupervised manor. Testing of sensorimotor performance and assessments of CMCT were performed on the same day, in the same room, and by the same unblinded investigator.

### Dropouts

One patient reported the development of pain in both upper limbs directly and progressively after an unrelated session of physical therapy (therapeutic climbing). One patient reported muscle soreness of the finger extensors the day after the first training session. One patient revealed a CMCT of 31 ms at the beginning and 28 ms at the end of the study and was removed as an outlier (a relapse occurred approx. 3 months prior to the study).

### Statistical analysis

The trained and untrained upper limbs’ performance and CMCT, as well as changes in CMCT, were compared using paired *t*-tests with a Bonferroni α correction to 0.05/4 = 0.0125 (one sided for the dominant upper limb). Further, a linear mixed-effects model (lme4 and lmerTest [32–34]) was computed for changes in CMCT by patient characteristics, baseline performance, and changes in PEG, 10 s TAP, and 10,000 TAP using RStudio (RStudio Inc.). In case of no significant random effects, a post hoc model of multiple linear regression was applied to predict changes in CMCT. The critical variance inflation factor (VIF) was set to 5.0. The intraclass correlation for the CMCT was computed for the nondominant upper limb. Effect sizes were computed as Cohen’s *d*, adjusted *R*^2^s, and *β*-weights (multiple linear regression), and proportional variance (linear mixed effects). α was set to 0.05.

## Results

**Fig. 1:**
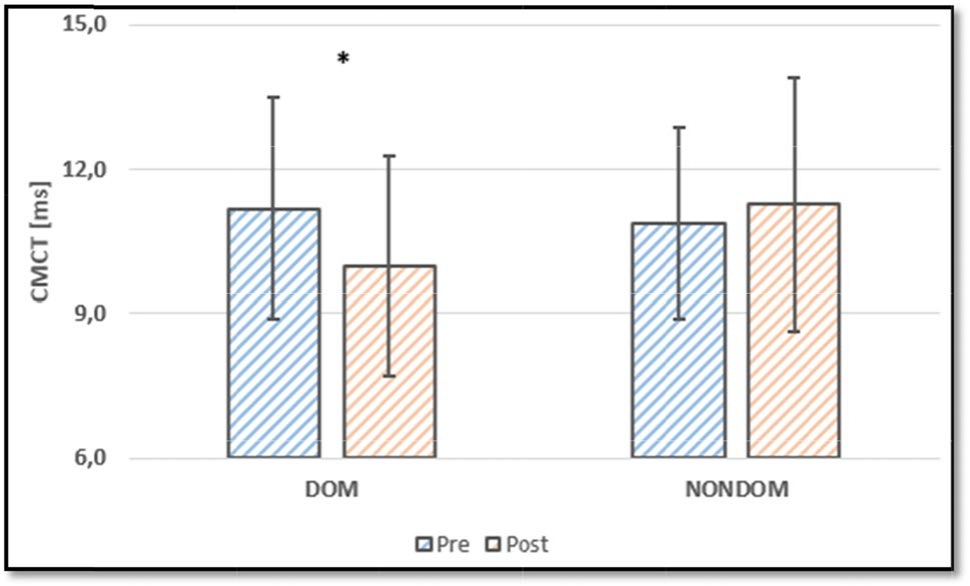
CMCT of the dominant (DOM) and nondominant (NONDOM) upper limb in session 1 (pre) and the last session (post). An * correspondents with statistical significance (*p* < 0.0125)

The 10 s TAP and PEG performance improved on the dominant side as well as the nondominant (except 10 s TAP of the nondominant side with a trend (*p* = 0.036)). CMCT only improved on the dominant side and was faster in 11 of 12 participants (92%) (Fig. 1). A post hoc analysis of peripheral conduction times (3 missing data points), revealed no significant changes for the dominant (*p* = 0.907) nor the nondominant upper limb (*p* = 0.576). Conduction times were 14.16 ms ± 1.41 ms (pretest) and 14.19 ms ± 1.27 ms (posttest) for the dominant and 14.30 ms ± 1.79 ms (pretest) and 14.44 ms ± 1.46 ms (posttest) for the nondominant upper-limb. The intraclass correlation coefficients for the peripheral conduction times were 0.92 (dominant side, *p* < 0.01) and 0.92 (nondominant side, *p* < 0.01). 10,000 TAP performance improved over the course of 5 days (by 4:29 min ± 0:58 min from 39:42 min ± 4:44 min to 35:13 min ± 4:48 min). All data are given in Table 2 (10 s TAP, PEG, and CMCT) and Table 3 (10,000 TAP and ∆CMCT).

**Table 2:** Performance of the dominant and nondominant hand at the beginning and end of the study

Upper limb	Time point and comparison	10 s TAP [Hz]	PEG [s]	CMCT [ms]
Dominant	*Pre*	5.54 ± 0.59	20.65 ± 4.44	11.19 ± 2.32
	*Post*	6.06 ± 0.71	18.27 ± 3.50	9.99 ± 2.29
	*p-value* *Cohen’s d*	0.001 0.79	0.009 0.60	0.001 0.52
Nondominant	*Pre*	4.68 ± 0.70	23.61 ± 5.70	10.87 ± 1.99
	*Post*	4.88 ± 0.69	21.59 ± 5.01	11.28 ± 2.65
	*p-value* *Cohen’s d*	0.036 *Nonsignificant*	0.005 0.38	0.259 *Nonsignificant*

**Table 3:** Ten thousand TAP performance at the beginning and end of the study and comparison of changes in CMCT between both upper limbs

Time point and comparison	10,000 TAP [Hz]	Upper limb and comparison	∆CMCT [ms]
*Pre*	4.25 ± 0.47	*Dominant*	− 1.20 ± 0.95
*Post*	4.80 ± 0.59	*Nondominant*	0.41 ± 2.12
*p-value* *Cohen’s d*	< 0.001 1.04	*p-value Cohen’s d*	0.011 0.98

**Fig. 2:**
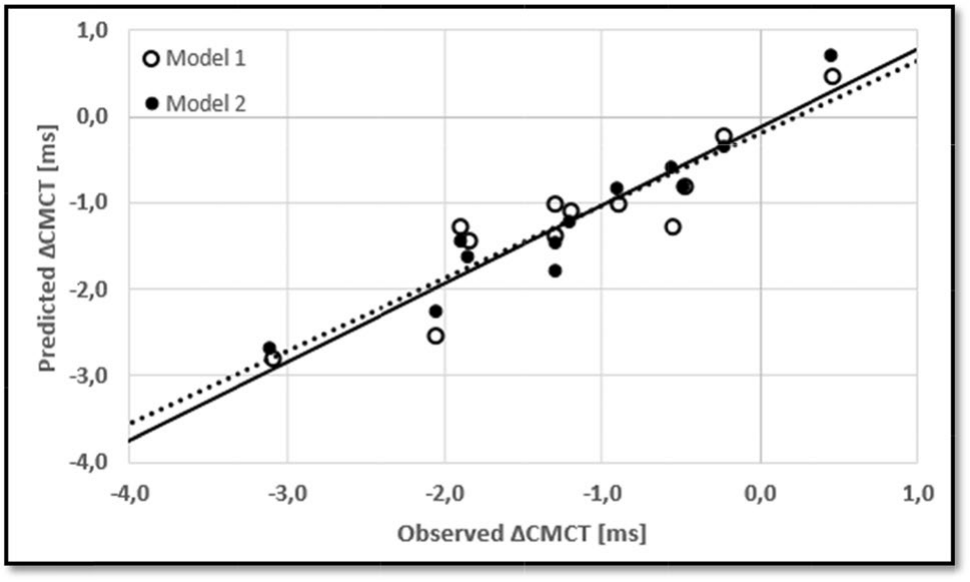
Observed and predicted changes in CMCT. Model 1 (circles and dotted line) had an *R*^2^_adjusted_ of 0.78 (*p* = 0.002). Model 2 (dots and solid line) had an *R*^*2*^_adjusted_ of 0.87 (*p* < 0.001)

There were no significant random effects for changes in CMCT, so we applied models of multiple linear regression. One resulting model (model 1) was significant with an *R*^2^_adjusted_ of 0.78 (*p* = 0.002, Table 4, Fig. 2). Factors were the 10,000 TAP performance at session 1, with higher frequencies predicting less reduction of the CMCT, the 10,000 TAP improvement over the training course in minutes with stronger improvements (e.g., 4 min equals a change from initially 38 to 34 min in the last session) predicting stronger reductions of CMCT, and interaction of the 10,000 TAP improvement (in minutes) and the initial difference of CMCTs between dominant and nondominant upper limb (i.e., CMCT_DOM_–CMCT_NONDOM_).

**Table 4:** Factors of the first and second multiple linear regression model for changes in CMCT

Model 1			
Factor	10,000 TAP at session 1	10,000 TAP improvement	10,000 TAP improvement × CMCT ∆ dom. vs. nondom at session 1 (interaction)
*β*-weight	0.53	− 0.56	− 0.62
VIF	1.10	1.01	1.10
*p*-value	0.008	0.004	0.003
Model 2			
Factor	BMI	10 s TAP at session 1	EDSS
β-weight	0.78	0.94	0.78
VIF	1.04	1.86	1.86
*p*-value	< 0.001	< 0.001	< 0.001

A second model of multiple linear regression (model 2) for changes in CMCT did not include any parameters of changes in performance (e.g., in 10,000 TAP). It resulted in an *R*^2^_adjusted_ of 0.87 (*p* < 0.001, Table 4, Fig. 2) and factors were the BMI (lower BMI predicting greater reductions in CMCT), 10 s TAP performance at session 1 (worse performance predicting greater reductions in CMCT), and the EDSS (lower EDSS grades predicting greater reductions in CMCT).

Further, single regressions showed three strong significant associations: First, changes in CMCT and changes in 10,000 TAP (*R*^2^ = 0.37, *p* = 0.035, greater improvements predicting greater reductions in CMCT). Second, changes in CMCT and FM (time since patient reported first manifestation) (*R*^2^ = 0.38, *p* = 0.031, longer time spans predicting greater reductions in CMCT). Third, changes in CMCT and BMI (*R*^2^ = 0.41, *p* = 0.026, lower BMIs predicting greater reductions in CMCT). Changes in 10 s TAP or PEG performance were not associated with changes in CMCT (*p*_10s TAP_ = 0.620, *p*_PEG_ = 0.722).

The intraclass correlation for the CMCT of the nondominant upper limb was 0.61 (*p* = 0.012). CMCT changes of both sides were not associated (*p* = 0.424), even after adjusting the CMCT of the dominant upper limb by the regression models (*p*_model 1_ = 0.181, *p*_model 2_ = 0.219).

## Discussion

In the reported study, we examined if changes in CMCT are reflected by the performance of a thumb tapping task in a sample of 12 MS patients. In order to evoke changes in CMCT, we introduced a highly repetitive thumb tapping over the course of 5 days. CMCT of the trained upper limb did significantly improve by an average of 1.2 ms in 11 of 12 patients. Although a quick tapping assessment of 10 s (10 s TAP) was not associated with those changes, changes in tapping performance over 10,000 repetitions (10,000 TAP) were able to explain 37% of the variance in changes in CMCT alone. A model of multiple linear regression of changes in CMCT even resulted in an *R*^2^_adjusted_ of 0.78, including three factors: The starting performance in the 10,000 TAP task, the absolute change (e.g., in minutes) in the 10,000 TAP task, and interaction of the 10,000 TAP absolute improvement and the difference in CMCT between dominant and nondominant upper-limb at the beginning of the experiment. A second model, not including parameters of changes of performance, even resulted in an *R*^2^_adjusted_ of 0.87.

One clear limitation that has to be stated ahead discussing our findings is the low intraclass correlation of CMCT (assessed by the untrained upper limb) of 0.61, which is in line with reports on the reliability of CMCT [31]. Changes in CMCT of the upper limb were not associated (*p* = 0.181–0.424), even after training adjustment of the dominant upper limb, so the chance of a systematic change could be expected very low (for instance: good day or bad day of patients concerning their CMCT, which is in line with recent findings on sensorimotor performance [5]).

We were able to predict changes in CMCT, but the prediction needed factors, which’ assessment would exceed the assessment time of CMCT by transcranial magnetic stimulation by far (model 1 and change in 10,000 TAP) or did not include any parameter of performance changes (model 2 and BMI or FM). Therefore, we are not able to present an economic or feasible surrogate. However, we were able to observe a set of interesting peculiarities:

First, CMCT strongly improved over the course of five days with an average of approx. 3 h of training volume (186 min ± 25 min). An underlying mechanism could be a restored conductivity of impaired nervous pathways or axonal sections [35, 36] or an enhanced conductivity at unimpaired axonal sections [35]. The factors of model 1—that a better 10,000 TAP performance at session 1 predicted less reduction in CMCT—rather pointed toward a restored conductivity, same as the interaction of 10,000 TAP improvements and initial CMCT difference between the upper limbs (with *less better* or even *worse* CMCT of the dominant side predicting a greater reduction in CMCT). If this training effect holds true in further studies, the wish for promotion of improving conductivity [15, 18, 19] could be granted in a quite simple and relatively quick way. Still, one has to consider alternative explanations for these findings. One key mechanism could have been a change in the excitability of the motor cortex. However, Koeneke et al. [22] observed no change of the motor threshold after extensive tapping training in their healthy sample. Also, higher excitability would not necessarily lead to lower CMCT (at least in this magnitude).

Second, changes in CMCT were significantly associated with FM. The longer the timespan since the notice of (potential) MS symptoms, the stronger the reduction in CMCT by training. An odd addition is missing associations with patients’ age, intake of DMT, EDSS, and type of MS or an impact of current smoking. Patrikios et al. [17] did, for instance, find a positive association between disease duration and remyelination. However, we describe the reaction to training, while Patrikios et al. [17] only observed cross-sectionally, so it remains unclear if the same phenomenon was described. *The more is broken, the more can be restored* would be supported by the impact of starting performance in model 1 (10,000 TAP and the interaction term) and in model 2 (10 s TAP). However, this seems to be contradicted by the impact of the EDSS in model 2, being in line with previously published evidence from inpatient rehabilitation (lower EDSS grades predicting better improvements in sensorimotor performance) [4]. The EDSS alone probably did not show a significant association with changes in CMCT due to its emphasis on gait in moderate and higher grades. Since longer timespans since the first manifestations were associated with higher reductions in CMCT, the EDSS could potentially represent the factor coined *unhealthy lifestyle* (from a perspective of neuroplasticity) [4] and the initial tapping performance of the specific sensorimotor impairment of the respective upper limb. This would support the assumption of *the more is broken*, *the more can be restored* and would underline the impact of the central nervous milieu as will be discussed in the following. Longer disease duration has been linked to normal levels of central nervous inflammation [37], so a central nervous milieu with lower inflammation could allow more plasticity. Third, changes in CMCT were significantly associated with the BMI (alone or in model 2). Lower BMIs predicting greater reductions in CMCT, or better: higher BMIs predict less reduction in CMCT due to training. Overweight and obesity have been linked to a chronic inflammatory state [38] and as Frischer et al. [37] observed: Inflammation and neurodegeneration in MS appear to be strongly associated. However, this does not automatically mean that a high BMI promotes neurodegeneration or attenuates neuroregeneration, but the strong link of BMI and changes in CMCT in our data could indicate the second to some extent.

Of particular interest for rehabilitation purposes is the observation of an improvement of motor performance on the nondominant side in 10 s TAP and PEG. A post hoc correlational analysis revealed no significant association between dominant and nondominant performance gains, so, alternatively to a skill transfer (Andree 2002), learning the task would be a potential explanation (reducing the observed potential training effects of the dominant upper limb) [10]. However, the reported intraclass correlation of PEG was 0.90 [12], so a skill transfer remains an option. This is supported by the performance gains in 10 s TAP, which has been shown to be a highly reliable test [5]. Still, the use of this within-subject control design demands caution when interpreting the effects of the control upper limb, since it has been repeatedly shown that, especially due to age of reduced function, the central nervous system is not working in a unilateral way [39].

One strong limitation of the current study should be mentioned. The investigator was not blinded. This should not strongly affect the derived CMCT, but could potentially have an effect on the sensorimotor performance. However, we argue that the internal consistency of our data indicates that a potential bias would be neglectable. All analyses were run after completing the dataset.

## Conclusion

CMCT was significantly reduced by 5 consecutive days of intensive and highly repetitive training. However, we were not able to present an economic (model 1) and feasible (model 2) surrogate for future studies on improvements of CMCT. The first model would take more effort than the TMS procedure, while the second model rather estimated the potential of CMCT change of the individual. The extent of changes in the CMCT appeared to be affected by (course) indicators of an inflammatory milieu (BMI, FM) and the extent of demyelination (motor performance and CMCT laterality). The performance of other tasks (10 s TAP and PEG) appeared to profit from training as well. An association between the amplitude of changes in CMCT and motor performance was visible in 10,000 TAP (but not 10 s TAP or PEG) and models of multiple linear regression revealed strong predictive capacity (*R*^2^_adjusted_ of 0.78 and 0.87). However, CMCT assessment by transcranial magnetic stimulation remains to be not replaceable by motor performance markers.

## Data Availability

On request from the corresponding author.
